# ﻿The millipede family Polydesmidae Leach, 1816 (Diplopoda, Polydesmida) from Vietnam, with a description of a new cavernicolous species

**DOI:** 10.3897/zookeys.1190.114958

**Published:** 2024-01-30

**Authors:** Anh D. Nguyen, Tam T. T. Vu, Katsuyuki Eguchi

**Affiliations:** 1 Institute of Ecology and Biological Resources, Vietnam Academy of Science and Technology, 18, Hoangquocviet Rd., Caugiay District, Hanoi, Vietnam Institute of Ecology and Biological Resources, Vietnam Academy of Science and Technology Hanoi Vietnam; 2 Graduate University of Science and Technology, Vietnam Academy of Science and Technology, 18, Hoangquocviet Rd., Caugiay District, Hanoi, Vietnam Graduate University of Science and Technology, Vietnam Academy of Science and Technology Hanoi Vietnam; 3 Graduate School of Science, Tokyo Metropolitan University, Minami-osawa 1-1, Hachioji, Tokyo 192-0397, Japan Tokyo Metropolitan University Tokyo Japan; 4 Department of International Health and Medical Anthropology, Institute of Tropical Medicine, 1-12-4 Sakamoto, Nagasaki University, Nagasaki, 852-8523, Japan Nagasaki University Nagasaki Japan

**Keywords:** Asia, cave fauna, COI sequence, diversity, new species, taxonomy

## Abstract

The millipede family Polydesmidae Leach, 1816 is reviewed in the scope of the Vietnamese fauna. The distribution of the species, *Polydesmusvietnamicus* Nguyen, 2009 is extended northward to Ha Giang Province. A new cavernicolous polydesmid, *Pacidesmustuachua***sp. nov.**, is described from two caves in northwestern Vietnam, representing the first record of the genus from Vietnam. Extensive illustrations and DNA barcodes are provided for both species, a revised key is presented to all 12 species of *Pacidesmus* Golovatch, 1991, as well as a key to all eight genera of Asian Polydesmidae.

## ﻿Introduction

The millipede family Polydesmidae Leach, 1816 is almost strictly Holarctic, consisting of more than 60 nominal genera or subgenera and nearly 400 species and subspecies (Hoffman 1980; Golovatch 1991). The family is mostly distributed in the Mediterranean area, whereas Central and East Asia, as well as the entire Nearctic Region, show lower generic and, to a lesser degree, species diversity (Golovatch 1991; Djursvoll et al. 2001). Only a few macropolydesmid genera are found in Asia and Indochina including *Epanerchodus* Attems, 1901, *Pacidesmus* Golovatch, 1991, *Polydesmus* Latreille, 1802–1803, *Nipponesmus* Chamberlin & Wang, 1953, *Gleninea* Turk, 1945, *Jaxartes* Verhoeff, 1930, *Schizoturanius* Verhoeff, 1931, and *Uniramidesmus* Golovatch & Mikhaljova, 1979 (Golovatch 1991; Geoffroy and Golovatch 2004; Mikhaljova 2004; Golovatch and Geoffroy 2006, 2014; Golovatch et al. 2007; Nguyen 2009; Antić et al. 2019; Liu and Golovatch 2020). In addition, a fossil species of the rather small western to central European genus *Propolydesmus* Verhoeff, 1895 has recently been described from the mid-Cretaceous amber of Myanmar (burmite, 99–100 Mya) (Su et al. 2023).

Vietnam has been known to harbour a rich fauna of millipedes with about 250 recorded species (Enghoff et al. 2004 and updated). Many new species have been discovered recently, including cave millipedes, e.g. *Hyleoglomerisalba*Nguyen et al., 2022 (Kuroda et al. 2022), *Tylopusnguyeni* Golovatch, 2019, and *Hylomussrisonchai* Golovatch, 2019 (Golovatch 2019), but only one polydesmid has hitherto been revealed: *Polydesmusvietnamicus* Nguyen, 2009 from Tam Dao National Park, Vinh Phuc Province (Nguyen 2009).

The present paper updates the knowledge of the family Polydesmidae in Vietnam, with the description of a new cavernicolous species found in northwestern Vietnam. An updated key to all 12 *Pacidesmus* species is also presented, as well as a key to all eight genera of Polydesmidae reported so far from Asia.

## ﻿Materials and methods

Millipede specimens were hand-collected from forests and caves in northern Vietnam and preserved in 85–90% ethanol. Morphological characters were investigated with an Olympus SZX16 stereomicroscope. Gonopods were dissected for morphological examination and photographed. Colour images were taken at various focal planes using a Nikon imaging system (Nikon-Br) coupled with a SMZ800N Nikon stereomicroscope. UV images were taken using a Sony a6000 digital camera attached to the aforementioned SMZ800N Nikon stereomicroscope under the UV flashlight Nichia Convoy. Images were stacked using Helicon Focus version 7.0 and assembled in Adobe Photoshop CS6. Scanning electron microscope (**SEM**) images were taken using the system Prisma E (ThermoFisher Scientific) in the Institute of Ecology and Biological Resources.

Total DNA was extracted using Qiagen DNeasy Blood and Tissue Kits. A 680-bp fragment of the mitochondrial gene, cytochrome c oxidase subunit I (**COI**), was amplified and sequenced using a pair of universal primers, LCO1490 and HCO2198 (Folmer et al. 1994). Polymerase chain reaction (**PCR**) conditions for amplification of the COI gene follow those of (Nguyen et al. 2017). The successfully amplified PCR products were sent to the FirstBase Company (Malaysia) for purification and sequencing. COI sequences were checked and confirmed using BLASTN 2.6.0+ search (Zhang et al. 2000) and registered for GenBank accession numbers.

Morphological terminology follows Liu and Golovatch (2020). All specimens reported here, including types, are deposited in the Institute of Ecology and Biological Resources (**IEBR**), Vietnam Academy of Science and Technology, Hanoi, Vietnam.

### ﻿Abbreviation

**IEBR-Myr** Institute of Ecology and Biological Resources, Myriapod collection.

## ﻿Results

### ﻿Taxonomy

**Order Polydesmida Pocock, 1887**

**Family Polydesmidae Leach, 1816**

#### ﻿Genus Polydesmus

Taxon classificationAnimaliaPolydesmidaPolydesmidae

Latreille, 1802–1803

C9FF403D-9589-5493-9877-74A2BC3E1CDB

##### Type species.

*Juluscomplanatus* Linnaeus, 1761, by monotypy.

##### Remarks.

*Polydesmus* is certainly the largest genus within the family Polydesmidae, with over 200 species and subspecies which mainly occur in Europe and the Mediterranean, west of the central Caucasus (Hoffman 1980; Djursvoll et al. 2001). A few species have been found in the Oriental Region, these being *Polydesmusjaponicus* Miyosi, 1956, *P.miyosii* Murakami, 1966, *P.tanakai* Murakami, 1970, and *P.tangonis* Murakami, 1973 from Japan; *P.moorei* Pocock, 1895 and *P.paludicola* Pocock, 1895 from eastern China; *P.liber* Golovatch, 1991 from Hong Kong, southern China (Golovatch 1991); and *P.vietnamicus* Nguyen, 2009, the only species of the genus from northern Vietnam (Nguyen 2009). The two old species of Pocock (1895) from mainland China are only provisionally to be assigned to *Polydesmus*, as both require revision. The distribution of *Polydesmus* seems to be amphi-Palaearctic (Golovatch 1991).

#### ﻿ Polydesmus vietnamicus

Taxon classificationAnimaliaPolydesmidaPolydesmidae

Nguyen, 2009

3F55FE8D-C96D-52C2-A3C2-952A0E5B9BF1

Figs 1 , 2 , 3 , 4 , 5 , 6 , 7 , 8

##### Materials examined.

Vietnam – Vinh Phuc Province • 1 ♂, 1 ♀; Vinh Phuc Province, Tam Dao National Park, near town; 1,000 m a.s.l.; 1 March 2005; Anh D. Nguyen leg.; natural secondary forest • 1 ♂; Vinh Phuc Province, Tam Dao National Park, on the way to Thac Bac waterfall; 1,000 m a.s.l.; 22 March 2005; Anh D. Nguyen leg.; bamboo forest, near stream • 1 ♂, 3 ♀s, 1 juvenile; Vinh Phuc Province, Tam Dao National Park, around the town; 900–1,000 m a.s.l.; 15–18 October 2010; Anh D. Nguyen leg.; mixed forest; IEBR-Myr 967 • 3 ♀♀; Tam Dao National Park, on way to Tam Dao 2; 1,100 m a.s.l.; 25 February 2017; Anh D. Nguyen leg.; natural forest; IEBR-Myr 604 – Ha Giang Province • 2 ♂♂, 1 ♀; Bac Me Natural Reserve, Lac Nong commune, Ban Khen; 22°45'30.8"N, 105°14'04.5"E; 11 December 2019; Anh D. Nguyen leg.; regenerated forest; IEBR-Myr 808.

**Figure 1.**
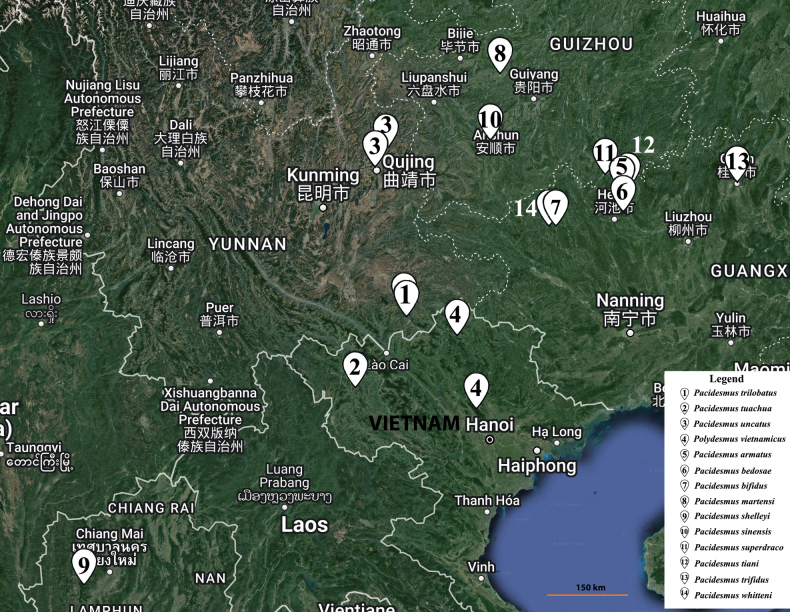
Records of polydesmid species in Vietnam and of all known *Pacidesmus* species.

##### Diagnosis.

A typical polydesmid with 20 body rings and three transverse rows of bosses with setae on metaterga. Gonopodal solenomere rather well developed, conspicuously shaped. Endomere elongate and strongly falcate, directed caudally, starting laterally and basally of recurvature point of seminal groove, set off from femorite by a sulcus, with a pair of strong teeth at about midway (**mt**). Seminal groove largely mesal, crossing the femorite diagonally, terminal lateral loop relatively short and turning around a distofemoral process (**ap**). Solenomere (**sl**) short, but evident and bifid.

**Figure 2.**
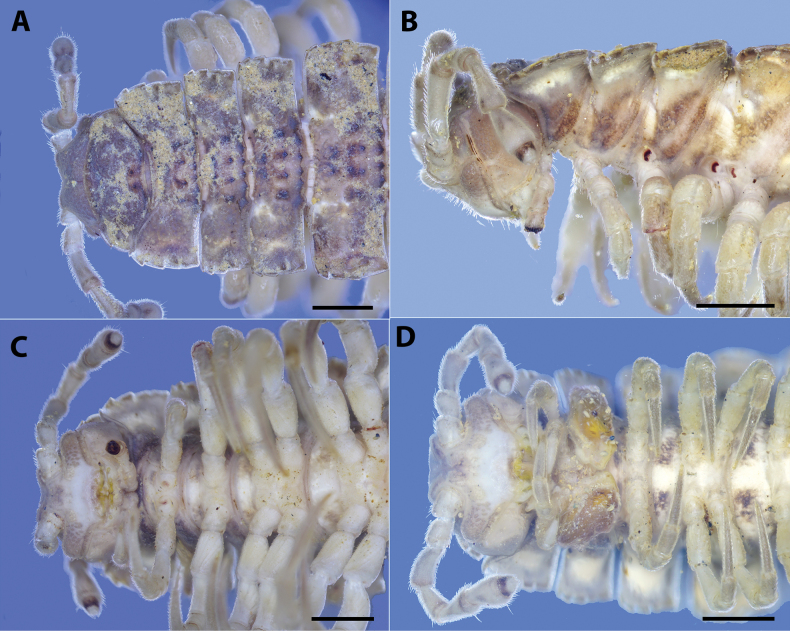
*Polydesmusvietnamicus* Nguyen, 2009 from Ha Giang Province (IEBR-Myr 808) **A–C** ♂. Anterior part of body **A** dorsal view **B** lateral view **C** ventral view **D** ♀, anterior part of body, ventral view. Scale bars: 1 mm.

The species differs from the morphologically particularly similar *Polydesmusliber* Golovatch, 1991 in being larger (33.0–38.4 mm vs 21.0–23.0 mm in length) and in the gonopod endomere (with a pair of teeth at about its midlength vs with two pairs of moderate teeth at 1/3 and 2/3 of its length).

It is particularly noteworthy that all East and Southeast Asian species undoubtedly belonging to *Polydesmus*, however few, share the symplesiomorphy of densely setose gonopod coxites, which contrasts with very poorly setose ones observed in the much more numerous western Palaearctic counterparts (Golovatch 1991).

##### DNA barcode.

The COI fragment (660 bp) was uploaded to GenBank with accession numbers PP118038 and PP118039. *Polydesmusvietnamicus* has a close COI identity to *Pseudopolydesmuspinetorum* (Bollman, 1888) (MT739870) and *Pseudopolydesmusserratus* (Say, 1821) (MT739862), with 89.8% (query coverage 83%) and 88.71% (query coverage 83%), respectively.

##### Remarks.

This species was previously known from only its type locality, Tam Dao National Park (Nguyen 2009). Currently, its distribution is extended northward to Ha Giang Province. There are no significant morphological variations between the type specimens and those samples collected in Ha Giang.

#### ﻿Genus Pacidesmus

Taxon classificationAnimaliaPolydesmidaPolydesmidae

Golovatch, 1991

7613815F-1882-5E36-A86C-43F289C982C3

##### Type species.

*Pacidesmusshelleyi* Golovatch, 1991, by original designation.

##### Remarks.

*Pacidesmus* contains 12 species found in southern China and northern Thailand (listed below). While the type species, *P.shelleyi* Golovatch, 1991, is known from forest litter at 2,200 m a.s.l. on Mount Doi Inthanon in northern Thailand (Golovatch 1991), the remaining species seem to be troglobionts restricted to caves in southern China, especially Guangxi and Guizhou provinces (Golovatch et al. 2010; Golovatch and Geoffroy 2014; Liu and Golovatch 2020).

#### ﻿ Pacidesmus tuachua sp. nov.

Taxon classificationAnimaliaPolydesmidaPolydesmidae

450DB17E-1A66-5A2B-8774-486FA1123F88

https://zoobank.org/CB8E9D35-3365-4416-81CA-589284A6D42F

Figs 1 , 9 , 10 , 11 , 12 , 13 , 14

##### Materials examined.

***Holotype*.** Vietnam • ♂; Dien Bien Province, Tua Chua District, Xa Nhe commune, Xa Nhe cave; 600 m a.s.l.; 21°52'37"N, 103°24'48"E; 12 April 2022; Anh D. Nguyen leg.; **IEBR-Myr 951H.**

**Figure 3.**
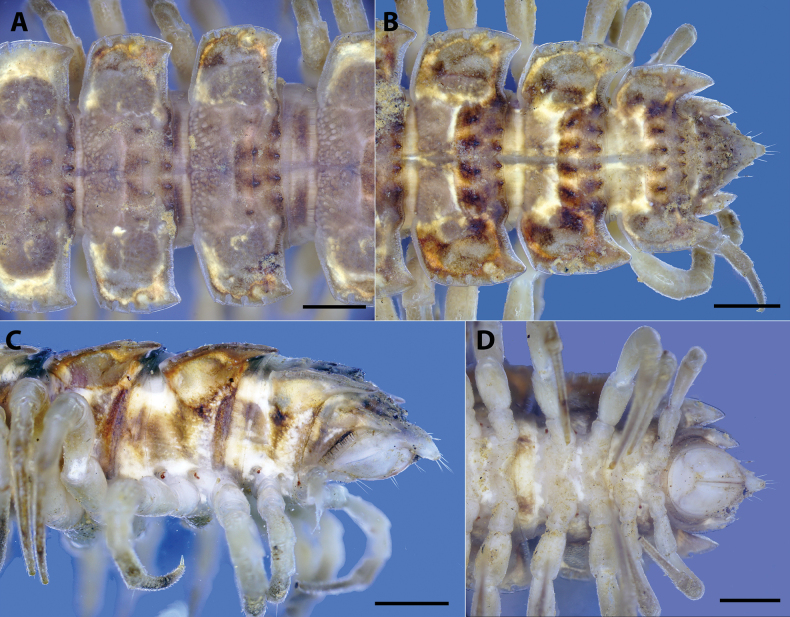
*Polydesmusvietnamicus* Nguyen, 2009 from Ha Giang Province (IEBR-Myr 808) ♂ **A** midbody segment 8–11, dorsal view **B** posterior part of body, dorsal view **C** posterior part of body, lateral view **D** posterior part of body, ventral view. Scale bars: 1 mm.

***Paratypes*.** Vietnam – Dien Bien Province • 5 ♂♂, 10 ♀♀; Tua Chua District, Xa Nhe commune, Kho Chua La cave; 600 m a.s.l.; 21°52'36.9"N, 103°24'47.9"E; 12 January 2021; Anh D. Nguyen leg.; IEBR-Myr 899 • 3 ♂♂, 4 ♀♀; Tua Chua District, Xa Nhe commune, Kho Chua La cave; 600 m a.s.l.; 21°52'36.9"N, 103°24'47.9"E; 12 January 2021; Anh D. Nguyen leg.; IEBR-Myr 900 • 4 ♂♂, 5 ♀♀; Tua Chua District, Xa Nhe commune, Kho Chua La cave; 600 m a.s.l.; 21°52'36.9"N, 103°24'47.9"E; 12 April 2022; Anh D. Nguyen leg.; IEBR-Myr 951P • 4 ♂♂, 5 ♀♀; Tua Chua District, Xa Nhe commune, Xa Nhe cave; 600 m a.s.l.; 21°52'37"N, 103°24'48"E; 12 April 2022; Anh D. Nguyen leg.; IEBR-Myr 952 • 2 ♂♂, 4 ♀♀; Tua Chua District, Xa Nhe commune, Kho Chua La cave; 600 m a.s.l.; 21°52'36.9"N, 103°24'47.9"E; 12 April 2022; Anh D. Nguyen leg.; IEBR-Myr 953.

**Figure 4.**
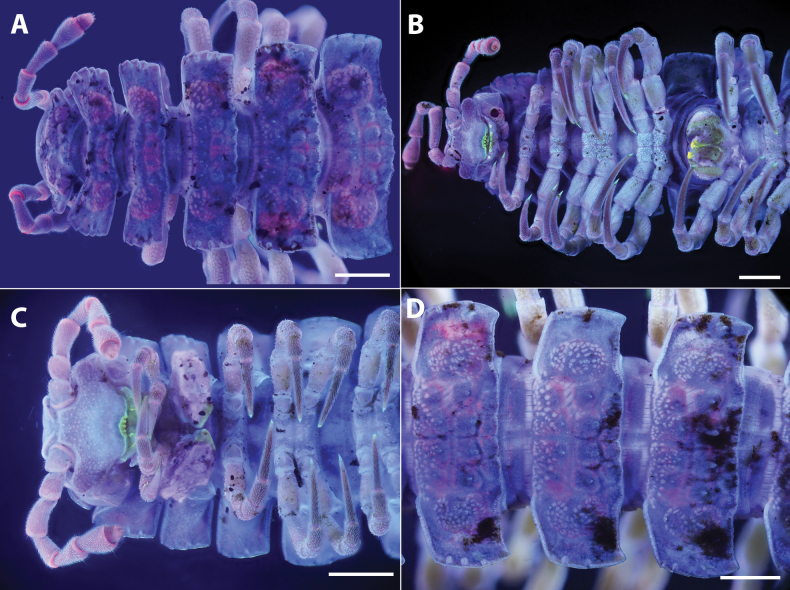
*Polydesmusvietnamicus* Nguyen, 2009 from Ha Giang Province (IEBR-Myr 808) ♂ and ♀ under the UV light **A** ♂, anterior part of body, dorsal view **B** ♂, anterior part of body, ventral view **C** ♀, anterior part of body, ventral view **D** ♂, midbody segment 8-10, dorsal view. Scale bars: 1 mm.

##### Diagnosis.

The new species can be distinguished from its congeners by a combination of the following features: unpigmented colouration, small size (midbody width <4.0 mm), head narrower than collum, absence of sphaerotrichomes, lateral budges on male prefemora, subfalcate gonopod telopodite, absence of exomere, endomere with an acute triangular process distally and a broad triangular process medially, and endomere tip slightly and unequally bifid.

**Figure 5.**
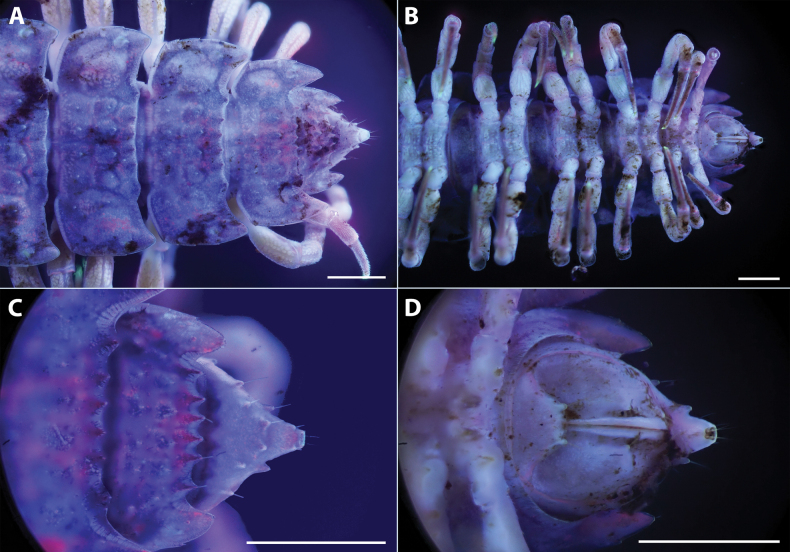
*Polydesmusvietnamicus* Nguyen, 2009 from Ha Giang Province (IEBR-Myr 808) ♂, under the UV light **A** posterior part of body, dorsal view **B** posterior part of body, ventral view **C** telson, dorsal view **D** telson, ventral view. Scale bars: 1 mm.

The species is truly cavernicolous, characterized by white or unpigmented colour and living within a cave. As a troglobiont species, it groups with all 12 troglobiont or troglophile congeners from China (Table 1). However, this species differs from all of these, except *P.bifidus* from the Hengli Xin Don Cave, Guangxi Province in the absence of an exomere and the gonopod telopodite showing no additional processes; the endomere also has two additional processes, and the tip of the endomere bears two tiny teeth. The new species is similar to *P.bifidus* in having a troglomorphic appearance, the absence of an exomere, and a bifid tip of the endomere, but it differs in having two tiny teeth at the tip of the endomere tip (vs two long processes in *P.bifidus*).

**Table 1.** List of all known species of the genus *Pacidesmus* Golovatch, 1991.

Species	Localities
*Pacidesmusarmatus* Golovatch, Geoffroy & Mauriès, 2010	China, Guangxi Prov., Huanjiang, Cave Xiao Lan Dong (Golovatch et al. 2010)
*Pacidesmusbedosae* Golovatch, Geoffroy & Mauriès, 2010	China, Guangxi Prov., Huanjiang, Cave Dong Tu Dong (Golovatch et al. 2010)
*Pacidesmusbifidus* Golovatch & Geoffroy, 2014	China, Guangxi Prov., Cave Hengli Xin Dong near Fengshan (Fengshan Xian) (Golovatch and Geoffroy 2014)
*Pacidesmusmartensi* Golovatch & Geoffroy, 2006	China, Guizhou Prov., Qianxi County, Hong Lin Town, Ishui Luo Dong Cave China, Guizhou Prov., Dafang County, Yangzhamba Village, Hei Dong Cave China, Guizhou Province, Qianxi County, Honglin Town, Jisha Village, I Dong Cave (Golovatch et al. 2007; Golovatch and Geoffroy 2006)
*Pacidesmusshelleyi* Goolovatch, 1991	Thailand, Chieng Mai Province, Doi Inthanon National Park (Golovatch 1991)
*Pacidesmussinensis* (Golovatch & Hoffman, 1989)	China, Guizhou Province, Ziyun County, Getuhe National Geopark, Suidao Dong Cave (Liu and Golovatch 2020) A cave in Guizhou Province and Cave Kaikou Dong, Zhenning County, Guizhou Province, China (Loksa 1960; Golovatch and Hoffman 1989; Chen and Meng 1990)
*Pacidesmussuperdraco* Golovatch, Geoffroy & Mauriès, 2006	Cave Laitai Dong, Libo County, Guizhou Province (Golovatch et al. 2007)
*Pacidesmustiani* Golovatch, Geoffroy & Mauriès, 2010	China, Guangxi Prov., Huanjiang, Cave Gang Lai Dong (Golovatch et al. 2010)
*Pacidesmustrifidus* Golovatch & Geoffroy, 2014	China, Guangxi Prov., Guilin County, Grotte des Squelettes (Golovatch and Geoffroy 2014).
*Pacidesmustrilobatus* Liu & Golovatch, 2020	China, Yunnan Province, Maguan County, Pojiao Town, Dayan Dong Cave China, Yunnan Province, Wenshan County, Liujing Town, Laozhai Village, I Dong Cave (Liu and Golovatch 2020).
*Pacidesmusuncatus* Liu & Golovatch, 2020	China, Yunnan Province, Qujing City, Zhanyi County, Tianshengqiao Dong Cave (Liu and Golovatch 2020)
*Pacidesmuswhitteni* Liu & Golovatch, 2020	China, Guangxi Zhuang Autonomous Region, Fengshan County, Jinya Town, Hangdong Village, I Dong Cave (Liu and Golovatch 2020)

**Figure 6.**
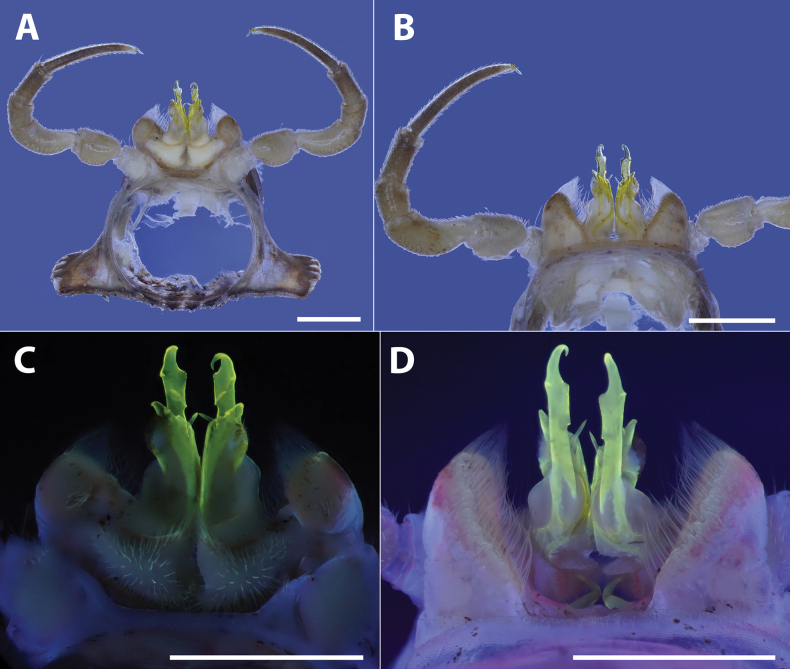
*Polydesmusvietnamicus* Nguyen, 2009 from Ha Giang Province (IEBR-Myr 808) ♂, segment 7 with gonopods under the normal (**A, B**) and UV (**C, D**) light **A, C** posterior views **B, D** anterior views. Scale bars: 1 mm.

The new species is assigned to *Pacidesmus* because of the following characters: the seminal groove starts mesally, then recurves laterad at the base of a particularly prominent endomere branch to enter an accessory seminal chamber that opens on a setose pulvillus; endomere bears additional processes.

**Figure 7.**
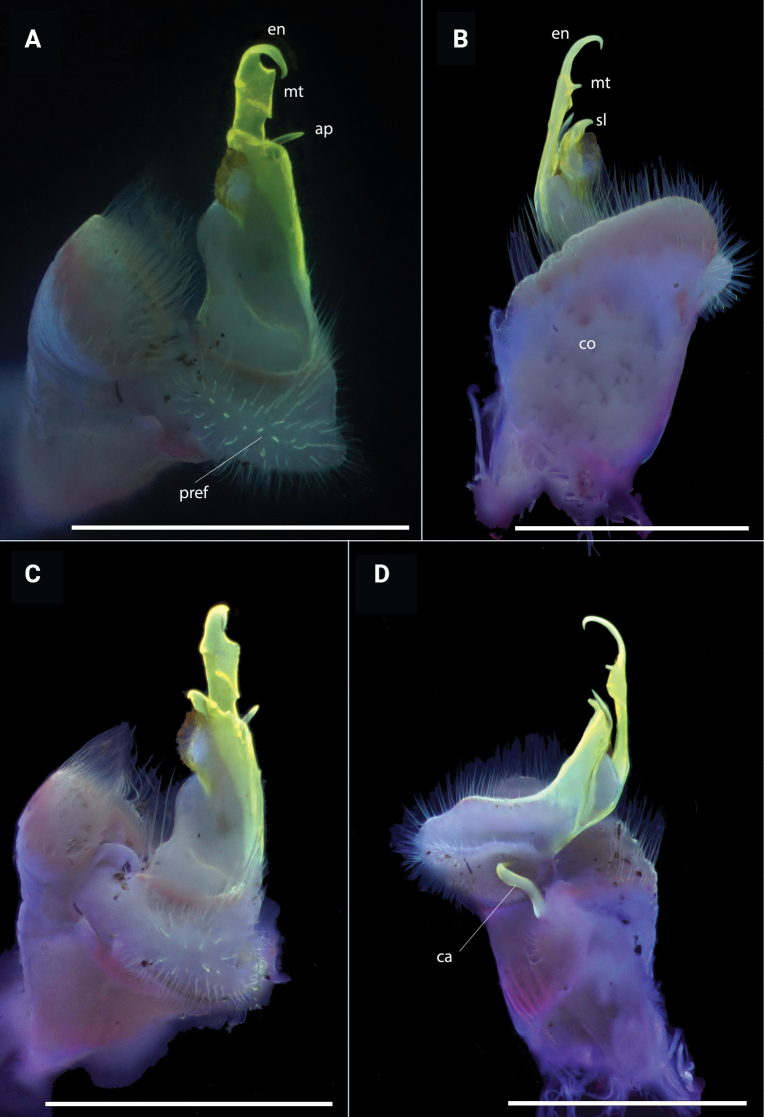
*Polydesmusvietnamicus* Nguyen, 2009 from Ha Giang Province (IEBR-Myr 808) ♂, right gonopod under the UV light **A** ventral view **B** lateral view **C** dorsal view **D** mesal view. Scale bars: 1 mm.

##### Etymology.

The specific epithet is treated as a noun in apposition and is based on the “Tua Chua” district where the two caves are located.

##### Description.

**Figure 8.**
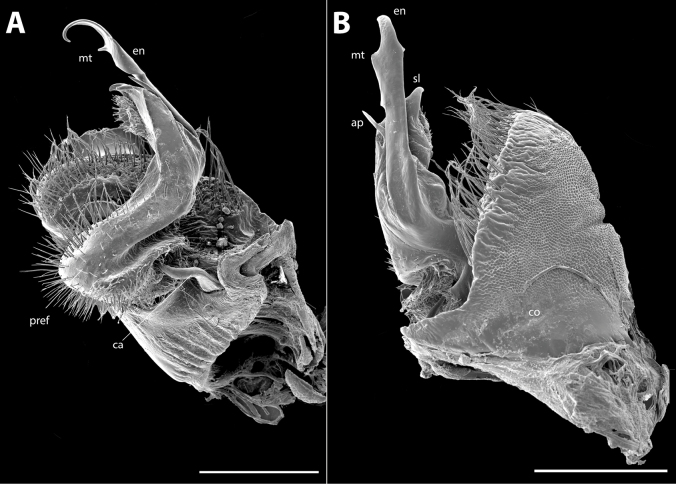
*Polydesmusvietnamicus* Nguyen, 2009 from Ha Giang Province (IEBR-Myr 808) ♂, right gonopod **A** mesal view **B** lateral view. Abbreviations: *co* = coxite; *pref* = prefemorite; *ca* = cannula; *en* = endomere; *sl* = solenomere; *mt* = midway teeth; *ap* = additional process. Scale bars: 0.5 mm.

**Figure 9.**
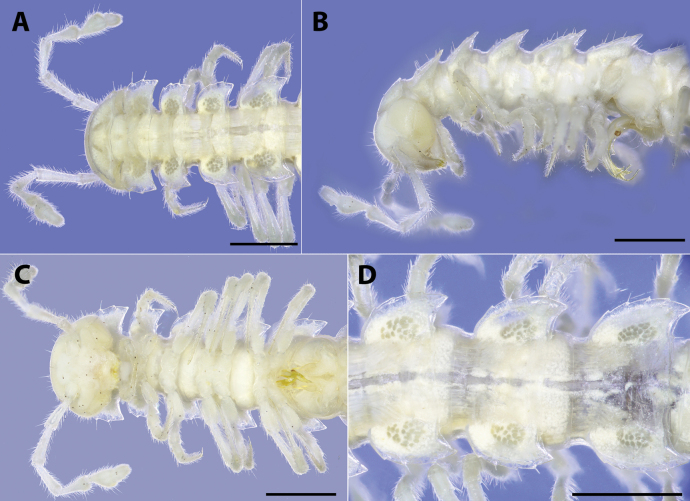
*Pacidesmustuachua* sp. nov., holotype ♂ (IEBR-Myr 951) **A** anterior part of body, dorsal view **B** anterior part of body, lateral view **C** anterior part of body, ventral view **D** segments 8–10, dorsal view. Scale bars: 1 mm.

**Figure 10.**
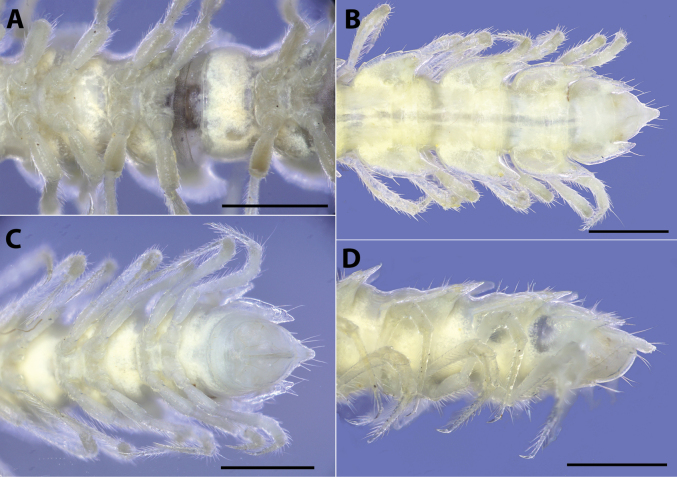
*Pacidesmustuachua* sp. nov., holotype ♂ (IEBR-Myr 951) **A** segments 8–10, ventral view **B–D** posterior part of body, dorsal, vental and lateral views, respectively. Scale bars: 1 mm.

**Figure 11.**
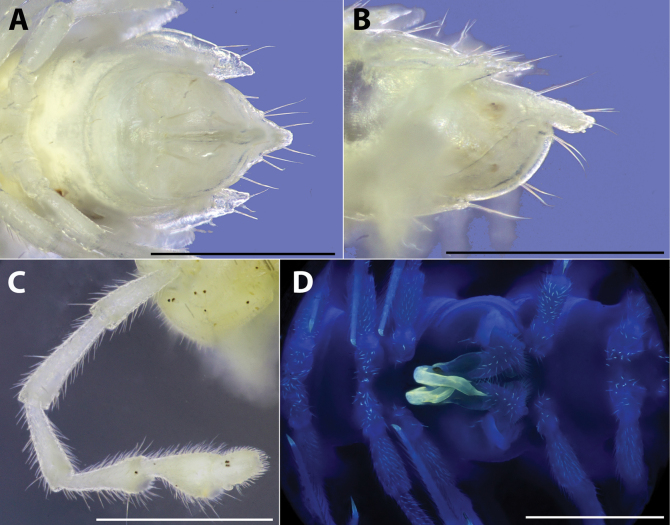
*Pacidesmustuachua* sp. nov., holotype ♂ (IEBR-Myr 951) **A** telson, ventral view **B** telson, lateral view **C** right antenna, anterior view **D** gonopods in situ under UV light, ventral view. Scale bars: 1 mm.

**Figure 12.**
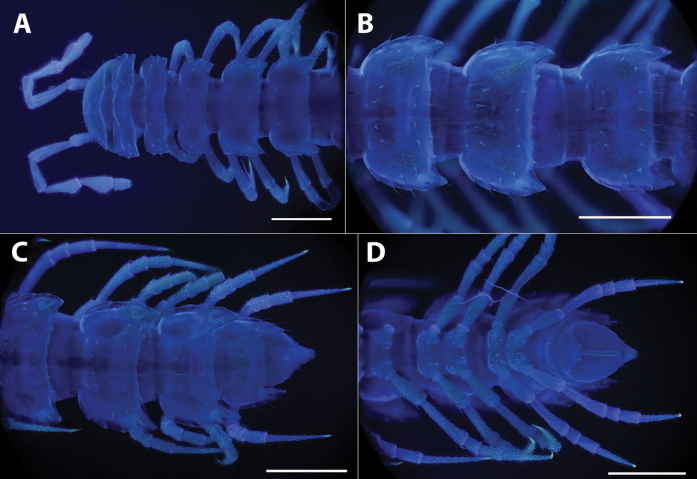
*Pacidesmustuachua* sp. nov., holotype ♂ (IEBR-Myr 951) **A** anterior part of body, under the UV light, dorsal view **B** segments 8–10, dorsal view **C** posterior part of body, dorsal view **D** posterior part of body, ventral view. Scale bars: 1 mm.

Holotype length ca 16.3 mm, width of midbody pro- and metazonae 1.0 mm and 1.5 mm, respectively. In width, head < collum < segment 3 = 4 < 2 < 5 = 15, thereafter body gradually tapering towards telson (Figs 9, 10, 12). Colouration in alcohol rather uniformly white (Figs 8, 9). Body with 20 segments. Antennae long and only slightly clavate, possibly reaching past segment 3 if stretching laterally; antennomere 3 longest, approximately 1.3× longer than subequal antennomeres 4–6; antennomeres 5 and 6 each with a small, compact, distodorsal group of bacilliform sensilla; antennomere 7 with a minute dorsoparabasal cone and a distodorsal group of microscopic sensilla (Figs 9A–C, 11C).

Paraterga (Figs 9, 10, 12A–C) strongly developed, set high, starting with collum, dorsum faintly convex; paraterga mostly weakly upturned above dorsum. Caudolateral corner of paraterga acute, postcollum ones extending increasingly past rear tergal margin, especially so in segments 16–18. All poreless segments with three incisions, all pore-bearing ones with four minute incisions at lateral margin. Front margins of metaterga narrowly bordered and forming distinct shoulders.

Ozopores evident, dorsal, located in front of posteriormost marginal incision of paraterga 5, 7, 9, 10, 12–13, 15–19.

Metatergal sculpture typical, poorly developed, obliterate, with three transverse rows of typical (= polydesmid), setigerous, polygonal bosses. Tergal setae short, slightly longer only on collum, simple, often obliterate. Stricture between pro- and metazona wide, shallow and nearly smooth. Limbus exceedingly thin, microdenticulate (Fig. 12A–C). Pleurosternal carinae absent.

Epiproct (Figs 11A, B, 12C, D) short, conical, pre-apical lateral papillae evident. Hypoproct (Figs 11A, 12D) subtriangular; distolateral setiferous knobs small, but distinct and well separated.

Sterna without modifications, but setose (Fig. 10A, C).

Legs generally long and slender, apparently slightly incrassate, approximately 1.7–1.8× as long as midbody height, densely setose, almost all setae simple, poorly branching setae with minute, distal, side branchlets only on slender prefemora, latter devoid of lateral bulges.

**Figure 13.**
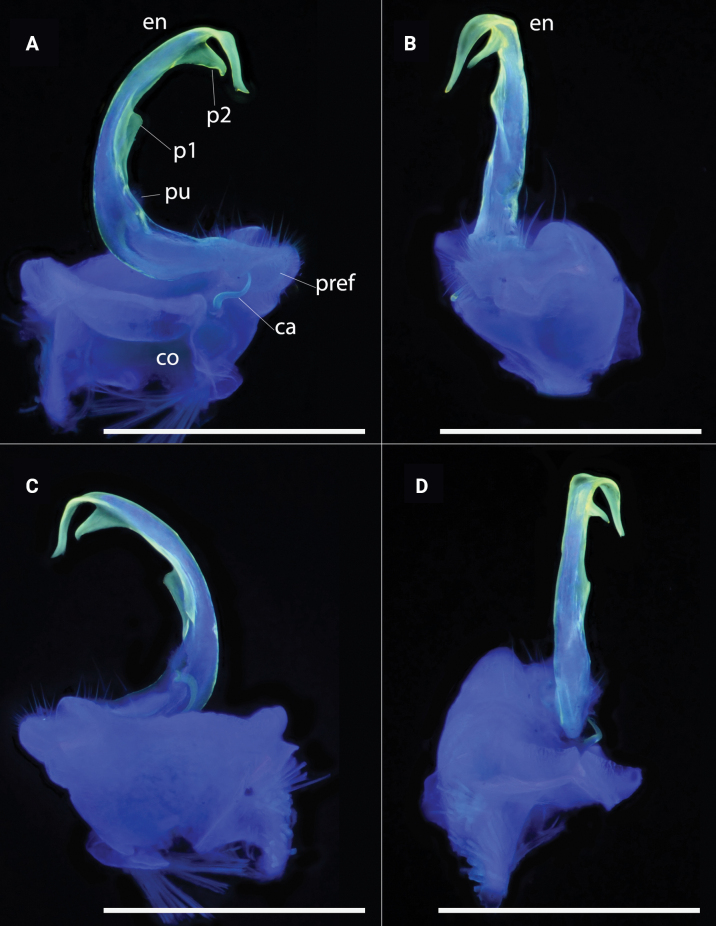
*Pacidesmustuachua* sp. nov., holotype ♂ (IEBR-Myr 951) right gonopod, under UV light **A** mesal view **B** ventral view **C** lateral view **D** dorsal view. Scale bars: 1 mm.

Gonopods (Figs 11D, 12, 13) characteristically subfalcate (vs suberect in all other congeners). with large, rectangular coxites (**co**), with a few long setae ventrally; a long, simple, flagelliform cannula (**ca**) as usual. Telopodite elongate, stout, strongly falcate or C-shaped; prefemorite (**pref**) densely setose; seminal groove starting mesally, then recurving laterad to run to the opening on a hairy pulvillus. Endomere (**en**) with two additional processes, a shorter, larger, broader triangular process at midlength (**p1**), and a longer, acuter, triangular process at ¾ length (**p2**). Tip of endomere unequally bifid, a longer and a shorter branch. Neither an exomere nor a clivus.

**Female.** Slightly larger than male, length ca 16.8 mm, width of pro- and metazona about 1.1 mm and 1.7 mm, respectively. Paraterga slightly less strongly developed. Legs unmodified, somewhat shorter and more slender. Vulvae highly elevated. Epigynal ridge low.

##### DNA barcode.

Two COI sequences (661 bp) were uploaded to the GenBank with the accession numbers PP118040 and PP118041. The new species has a close COI identity to *Epanerchoduskoreanus* (NC051495) at 88.72% (query coverage 97%).

##### Habitat.

This species is to be considered a true troglobiont because it shows the typical morphological features of a cave-dweller. It was collected exclusively in the dark zone of the caves as described below. Kho Chua La and Xa Nhe caves are both located close together, approximately 500 m in distance. These caves are at the centre of the Xa Nhe commune, Dien Bien Province, northwestern Vietnam. The two caves are tunnel-like: they are high (15–20 m), wide (15–20 m), and long (1,000–1,500 m). The floor is mainly wet, with clay, and some small pools. Several other millipede species have been found in these caves, including *Glyphiulus* sp. (Spirostreptida, Glyphiulidae) and *Eutrichodesmus* sp. (Polydesmida,: Haplodesmidae). The new species was found >1000 m from the entrance.

Kho Chua La and Xa Nhe caves are located on the Tua Chua karst plateau in northeastern Dien Bien Province, northwestern Vietnam. The natural area is about 68,414 ha, and 70% of this area is composed of limestone mountains, which are known for their layers of majestic rugged rock and unique natural landscape. The karst region contains many stunning and well-known caves, such as Kho Chua La, Tham Khem, Hau Chua, Xa Nhe, and Pe Rang Ki (Nguyen et al. 2022). Furthermore, the Tua Chua karst plateau of northwestern Vietnam is close to the Yunnan and Sichuan karst regions of southwestern China. Given this, it is not surprising to discover the genus *Pacidesmus* in northwestern Vietnam. The distance between *Pacidesmustuachua* sp. nov. and *Pacidesmustrilobatus* Liu & Golovatch, 2020 from Guangxi Province, China, is about 150 km northeast–southwest (Fig. 1).

**Figure 14.**
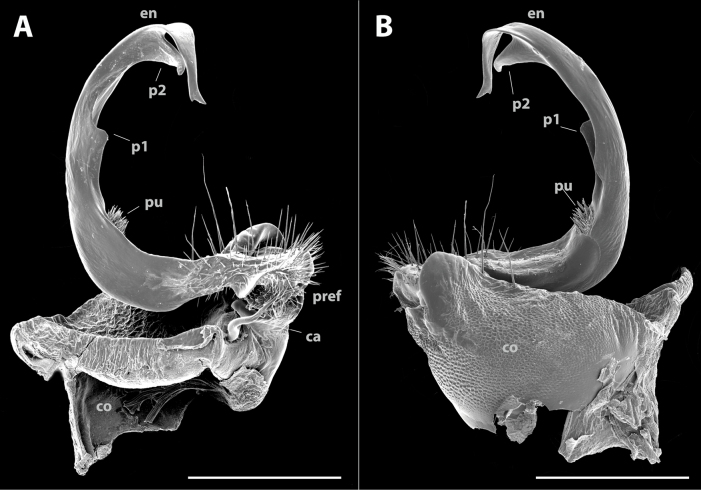
*Pacidesmustuachua* sp. nov., holotype ♂ (IEBR-Myr 951) right gonopod **A** mesal view **B** lateral view. Abbreviations: *co* = coxite; *pref* = prefemorite; *ca* = cannula; *en* = endomere; *pu* = puvillus; *p1* = first process; *p2* = second process. Scale bars: 0.5 mm.

##### Remarks.

While there remains a noticeable geographical gap between the mountainous northern Thailand species and the troglobionts of southern China, the discovery of a new species in northern Vietnam partially fills this gap. Like the more eastern species, the new species is also troglobiotic, and whether these species should be classified in a distinct genus related to a more restricted *Pacidesmus* (including only the type species) needs exploration.

The discovery of a new species marks the first record of the genus *Pacidesmus* in Vietnam.

### ﻿Identification key to species of the genus *Pacidesmus* Golovatch, 1991

Modified and updated from Golovatch et al. (2010).

**Table:** 

1	Sternal cones between ♂ legs 6 and 7 for accommodation of distal parts of gonopods present. Epigean and high-montane from northern Thailand	***P.shelleyi***
–	No such sternal modifications. Cavernicoles from southern China and northern Vietnam	**2**
2	Gonopod exomere absent	**3**
–	Gonopod exomere present	**4**
3	Gonopod telopodite suberect. Endomere tip clearly, deeply and narrowly bifid; endomere rather stout, not carrying any processes. Guangxi	***P.bifidus***
–	Gonopod telopodite subfalcate. Endomere tip slightly bifid; endomere slender, carrying two additional processes, a shorter, larger and broader triangular one at midlength, and a longer, acuter triangular one at ¾ length. Northern Vietnam	***P.tuachua* sp. nov.**
4	Gonopod exomere without process at base. Endomere tip bifid	**5**
–	Gonopod exomere with a process at base. Endomere tip either trifid or unifid	**6**
5	Gonopod endomere less stout. Body length 23–24 mm, width of midbody pro- and metazona 1.5–1.6 and 2.5–2.7 mm, respectively. Paraterga upturned above dorsum only on rings 1–5. Guangxi	***P.bedosae***
–	Gonopod endomere stouter. Body length 28–30 mm, width of midbody pro- and metazona 1.4–1.7 and 2.8–3.2 mm, respectively. Paraterga upturned above dorsum until ring 17 (♂) or 14 (♀). Guizhou	***P.superdraco***
6	Gonopod endomere rather stout, tip either unifid or trifid. Exomere small	**7**
–	Gonopod endomere slender, tip unifid. Exomere large	**8**
7	Only paraterga 1–4 evidently upturned above dorsum. Gonopod endomere slender, subfalcate, carrying a small tooth distally on median surface; tip unifid	***P.tiani***
–	Paraterga upturned above dorsum starting with paraterga 2. Gonopod endomere stouter, suberect, not carrying any processes. Tip trifid	***P.trifidus***
8	Endomere with two teeth	**9**
–	Endomere with either one tooth or three teeth	**10**
9	Caudolateral corners of paraterga strongly triangular. Gonopod exomere larger, finger-shaped	***P.whitteni***
–	Caudolateral corners of paraterga narrowly rounded to pointed. Gonopod exomere smaller and unciform	***P.armatus***
10	Endomere with one tooth	**11**
–	Endomere with three teeth	**12**
11	All paraterga clearly upturned above dorsum. Endomere rather long and strongly twisted	***P.uncatus***
–	Only anterior paraterga upturned above dorsum. Endomere much shorter and subfalcate	***P.sinensis***
12	Endomere slender and flagelliform, carrying a small denticle frontally at base. Exomere with a large membranous process at base	***P.martensi***
–	Endomere long and slender, carrying three lobes. Exomere with a short spiniform process at base	***P.trilobatus***

## ﻿Discussion

Beyond southern China and Southeast Asia, there are eight macropolydesmid genera occurring in Asia, mainly in Central Asia. The main differences between those genera are presented in Table 2 below.

**Table 2.** Morphological diagnoses and distribution of all eight macropolydesmid genera in Asia.

No.	Genus	Diagnosis	Distribution
1	*Schizoturanius*	Body small, strongylosomoid (= without prominent paraterga), moniliform; paraterga narrow, only seldom incised laterally; gonopods falcate and bifid distally; an accessory seminal chamber present; gonopod femorite carrying a characteristic process (Mikhaljova 2004)	Ten species in Central Asia and Ukraine, Asian part of Russia, Kazakhstan, northwestern China (Mikhaljova 2004; Nefediev 2023)
2	*Uniramidesmus*	Body small (usually ca 10 mm long); head covered with dense minute hairs; antennomeres 5–7 each with a small field of tiny bacilli dorsally; metaterga rather convex; paraterga small or medium-sized and set below dorsum, with marginal incisions; metatergal polygonal sculpture ranging from well-developed to poorly-developed; metatergal setae pointed; sphaerotrichs present or absent; gonopods slender, strongly falcate to coiled caudally, relatively simple, in situ crossing each other; seminal groove with a loop parabasally; accessory seminal chamber absent; opening of seminal groove subterminally to terminally on a bare to more or less pubescent pulvillus (Mikhaljova 2004, 2017)	Ten species in central Asia and Asian part of Russia (Mikhaljova 2004, 2017)
3	*Jaxartes*	Body small (usually ca 10 mm long); metaterga with bosses/tubercles with bacilliform or trichoid setae; paraterga clearly incised laterally; four distal ♂ podomeres with ventral sphaerotrichomes; gonopod coxite without outgrowths other than cannula; the gonotelopodite particularly slender, suberect, with the endomere being considerably longer than a ventrally fringed process (if present at all), also bearing a parabasal tooth and a subtruncate apex, basally with a very evident hairy pulvillus, but no distinct accessory seminal chamber (Antić et al. 2019)	Twelve species in Central Asia (Antić et al. 2019)
4	*Epanerchodus*	Gonopod endomere mostly absent, rarely present as only a more or less rudimentary structure, while the seminal groove after the recurvature point still makes a long way basad to debauch into a prominent, simple-haired, accessory seminal chamber placed at the bottom of a profound parabasal cavity in the telopodite (Liu and Golovatch 2018; Golovatch 2021).	120+ species, largely Palaearctic in distribution, mainly from Japan (East Asia) to the western part of China, from Mongolia (Central Asia) in the north to southern China and the Himalaya of Nepal in the south (Liu and Golovatch 2018; Golovatch 2021)
5	*Pacidesmus*	Body large, up to 30 mm long. Paraterga broad, slightly incised laterally. Metaterga with bosses with setae. Gonopod endomere variable, from relatively short, stout and bifid to long, slender and rather simple; exomere absent or supplied with an outgrowth (Golovatch et al. 2010; Liu and Golovatch 2020)	Twelve species in southern China and northern part of Southeast Asia (Golovatch et al. 2010; Liu and Golovatch 2020)
6	*Nipponesmus*	Body size large (up to 20 mm in length). Paraterga broad. Gonopod endomere with conspicuous comb of setae or slender teeth. The seminal groove running mostly mesally to recurve neatly between exomere and endomere, then to debauch somewhat basally into a prominent hairy pulvillus which also beset with the same peculiar trichome, and is devoid of an accessory seminal chamber (Golovatch et al. 2011)	Three species in Japan and Taiwan. (Golovatch et al. 2011)
7	*Gleninea*	Body small to large size (up to 16 mm in length). The third pair of ♂ legs only slightly thickened. Antenomere 5-6, each with a s mall, compact, distodorsal group of bacilliform sensilla. Lateral side of paraterga strongly serrated, 5-6 small, sharp teeth. Gonopod exomere simple, subfalcate; endomere with distal part carrying numerous or several spine-like hairs or strong, sometime curved spines. Accessory seminal chamber present (Golovatch and Geoffroy 2014)	Seven species in the Himalaya of India, Nepal, Bhutan, and China (Golovatch and Geoffroy 2014)
8	*Polydesmus*	Body size medium to large. Paraterga usually wide. Gonopod solenomere absent to rather well developed, sometimes conspicuously shaped. Exomere from short and slightly curved to very long and strongly falcate, mostly uniramous, directed caudally, starting laterally or apically and basally of recurvature point of seminal groove. Seminal groove, largely mesal; terminal laterad loop relatively short and turning around a distofemoral process (Djursvoll et al. 2001)	About 200 species distributed mainly in the Mediterranean; a few species in East Asia and northern Vietnam (Djursvoll et al. 2001)

*Pacidesmustuachua* sp. nov. is distinguished from members of *Nipponesmus* by having a gonopod endomere; the gonopod telopodite has neither a comb of setae nor slender teeth (vs without endomere, with a conspicuous comb of setae or slender teeth) (Golovatch et al. 2011). The new species differs from members of *Gleninea* in the absence of a gonopod exomere, the gonopod endomere is with only two additional processes, the absence of an accessory seminal chamber (vs the presence of a gonopod exomere, the distal part of the endomere carrying numerous or several spine-like hairs or strong, sometime curved spines, and the presence of an accessory seminal chamber) (Golovatch and Geoffroy 2014).

The new species is also distinguished from *Schizoturanius* and *Uniramidesmus* species by its larger size (16.3 mm vs less than 10.0 mm). The new species could possibly be assigned to the genus *Uniramidesmus* based on the simple, slender, falcate gonopod; however, *Uniramidesmus* species are all much smaller (<10.0 mm in length), the gonopods are strongly falcate to coiled caudally and cross each other when in situ; the opening of the seminal groove is subterminal to terminal on a bare to more or less pubescent pulvillus (Mikhaljova 2004). On the contrary, the new species is far larger in size (ca 16.3 mm in length), the gonopods in situ are well separated from each other, and the opening of the seminal groove is on a typical hairy pulvillus.

Compared to *Schizoturanius* (Mikhaljova 2004), *Pacidesmustuachua* sp. nov. differs well-developed paraterga (vs a strongylosomoid, moniliform body with narrow to almost missing paraterga, which are mostly smooth and only seldom laterally incised); also, the gonopod is without an accessory seminal chamber (vs with an accessory seminal chamber) and there is no gonopod femoral process (vs with a characteristic femoral process). Finally, the new species can hardly be assigned to the genus *Jaxartes*, which is confined to Central Asia, due to its larger body size (16 mm long), the slender, strongly falcate gonopods without an accessory seminal chamber. On the contrary, the genus *Jaxartes* is diagnosed by its small body (usually ca 1 cm long); the metaterga show bosses or tubercles with bacilliform or trichoid setae, the paraterga are clearly laterally incised, there are four distal male podomeres with ventral sphaerotrichomes, the gonopod coxite is without outgrowths apart from the typical cannula, and the gonotelopodite is particularly slender, suberect, and with the endomere being considerably longer than a ventrally fringed process (if present at all); also, the endomere bears a parabasal tooth and has a subtruncate apex, basally with a very evident hairy pulvillus, but there is no distinct accessory seminal chamber (Antić et al. 2019).

*Pacidesmustuachua* sp. nov. can hardly be placed in *Epanerchodus* or *Polydesmus* because its paraterga are relatively narrow, the seminal groove starts mesally, as usual, then is recurved laterad at the base of a particularly prominent endomere branch to enter an accessory seminal chamber that opens on a setose pulvillus, and the endomere bears additional processes.

The strongly sigmoid gonopodal telopodite in *P.tuachua* sp. nov is somewhat unusual in comparison to that in other *Pacidesmus* species. This difference may suggest a new genus; however, it currently seems best assigned to *Pacidesmus* based on the above discussion. It is noteworthy that most *Schizoturanius* or *Polydesmus* spp. likewise show only slightly curved gonopod telopodites, but relatively few species in these genera are so strongly sigmoid.

To support further study of Polydesmidae in Vietnam and Southeast Asia, an identification key to macropolydesmid genera occurring in Asia is provided:

### ﻿An identification key to macropolydesmid genera in Asia

Based on Turk (1945), Golovatch (1991), Djursvoll et al. (2001), Mikhaljova (2004), Golovatch et al. (2011), Golovatch and Geoffroy (2014), Antić et al. (2019).

**Table:** 

1	Gonopods without an accessory seminal chamber (*Uniramidesmus*, *Nipponesmus*, *Jaxartes*)	**2**
–	Gonopods with an accessory seminal chamber (*Schizoturanius*, *Pacidesmus*, *Gleninea*, *Polydesmus*, *Epanerchodus*)	**4**
2	Body moniliform, paraterga narrower. Seminal groove running mostly mesally to recurve neatly between exomere and endomere. Gonopod endomere distally with abundant bacilliform filaments	***Nipponesmus***
–	Body not moniliform, paraterga broader. Seminal groove running entirely mesally. Gonopod endomere distally without abundant bacilliform filaments	**3**
3	Seminal groove opening subterminally to terminally on a bare to more or less pubescent pulvillus	***Uniramidesmus***
–	Seminal groove opening on a distinct, ventral, hairy pulvillus	***Jaxartes***
4	Body moniliform, paraterga narrow. Loop of seminal groove distal	***Schizoturanius***
–	Body not moniliform, paraterga broad. Loop of seminal groove not distal	**5**
5	Paraterga strongly serrated or incised laterally. Gonopod endomere in distal part carrying numerous or several spine-like hairs or strong, sometime curved spines	***Gleninea***
–	Paraterga not strongly serrated/incised laterally. Gonopod endomere without numerous, mostly strong, sometimes curved spines or bacilli or setae	**6**
6	Paraterga narrower. Seminal groove starting mesally, then recurving laterad at the base of a particularly prominent endomere branch to enter an accessory seminal chamber that opens on a setose pulvillus; endomere carrying additional processes	***Pacidesmus***
–	Paraterga wider. Seminal groove largely running mesally. Gonopod endomere absent	7
7	An endomere absent. Exomere from short and slightly curved to very long and strongly falcate, mostly uniramous, directed caudally, starting laterally or apically and basally of recurvature point of seminal groove	***Polydesmus***
–	An endomere mostly absent, but rather rarely present as only a more or less rudimentary structure, while the seminal groove after the recurvature point still makes a long way basad to debauch into a prominent, simple-haired, accessory seminal chamber placed at the bottom of a profound parabasal cavity in the telopodite	***Epanerchodus***

## ﻿Conclusion

Two polydesmid genera and species are presently known to occur in Vietnam: *Polydesmusvietnamicus* Nguyen, 2009 and *Pacidesmustuachua* sp. nov. *Polydesmusvietnamicus* is an epigean, forest-dwelling species, while *Pacidesmustuachua* is troglobiotic. The diversity of polydesmids in Vietnam is potentially greater given the number of species in other southern Asian regions. The paucity of species is either due to some yet-unknown historical, evolutionary phenomenon or, more likely, reflects insufficient sampling. More extensive surveys are needed to more fully clarify the diversity and biogeography of polydesmids in Southeast Asia.

## Supplementary Material

XML Treatment for Polydesmus

XML Treatment for Polydesmus vietnamicus

XML Treatment for Pacidesmus

XML Treatment for Pacidesmus tuachua

## ﻿Additional information

### Author ORCIDs

Anh D. Nguyen https://orcid.org/0000-0001-9273-0040

Tam T. T. Vu https://orcid.org/0000-0003-1145-975X

Katsuyuki Eguchi https://orcid.org/0000-0002-1054-1295

### Data availability

All of the data that support the findings of this study are available in the main text.
